# Novel vaccination approaches to prevent tuberculosis in children

**DOI:** 10.1186/s41479-016-0020-z

**Published:** 2016-11-24

**Authors:** James A. Triccas, Claudio Counoupas

**Affiliations:** 1 Microbial Pathogenesis and Immunity Group, Department of Infectious Diseases and Immunology, Sydney Medical School, University of Sydney, Level 5, Charles Perkins Centre D17, Sydney, NSW 2006 Australia; 2 Tuberculosis Research Program, Centenary Institute, University of Sydney, Sydney, NSW Australia; 3 Sydney Medical School and the Marie Bashir Institute for Infectious Diseases and Biosecurity, University of Sydney, Sydney, NSW Australia

**Keywords:** Tuberculosis, Vaccine candidates, Bacille Calmette-Guérin, BCG, Immune response, Clinical trials

## Abstract

Pediatric tuberculosis (TB) is an underappreciated problem and accounts for 10 % of all TB deaths worldwide. Children are highly susceptible to infection with *Mycobacterium tuberculosis* and interrupting TB spread would require the development of effective strategies to control TB transmission in pediatric populations. The current vaccine for TB, *M. bovis* Bacille Calmette-Guérin (BCG), can afford some level of protection against TB meningitis and severe forms of disseminated TB in children; however, its efficacy against pulmonary TB is variable and the vaccine does not afford life-long protective immunity. For these reasons there is considerable interest in the development of new vaccines to control TB in children. Multiple vaccine strategies are being assessed and include recombinant forms of the existing BCG vaccine, protein or viral candidates designed to boost BCG-induced immunity, or live attenuated forms of *M. tuberculosis*. A number of these candidates have entered clinical trials; however, no vaccine has shown improved protective efficacy compared to BCG in humans. The current challenge is to identify the most suitable candidates to progress from early to late stage clinical trials, in order to deliver a vaccine that can control and hopefully eliminate the global threat of TB.

## Background

Tuberculosis (TB), caused by the intracellular bacterial pathogen *Mycobacterium tuberculosis*, remains a major cause of mortality and morbidity worldwide. Annually there are an estimated 9.6 million new cases of clinical TB and 1.5 million deaths, the majority occurring in South-East Asia [1]. The spread of TB is fueled by the human immunodeficiency virus (HIV)/acquired immune deficiency syndrome (AIDS) pandemic, the emergence of multi-drug resistant strains and socio-political disruption to health services. TB is a chronic infection and is generally considered a diseases of adulthood, however pediatric TB is an underappreciated problem. Infants under the age of two have the highest risk of contracting TB [2]. One million children contracted TB in 2014 and approximately 10 % of all TB deaths were in children [1]. The current TB vaccine, *M. bovis* Bacille Calmette-Guérin (BCG) can provide some protection against severe forms of pediatric TB, but its efficacy is variable, particularly against pulmonary disease in infants and adults. This review discusses the novel strategies being used to develop new TB vaccines, provides an overview of the candidates in clinical trials and outlines the challenges of introducing a new TB vaccine into existing childhood vaccination schedules.

## Vaccination against TB: the current state of play

BCG, an attenuated live form of *M. bovis*, has been in use since the early 1920s and is the only approved vaccine for the control of TB in humans. BCG can afford some level of protection, particularly against TB meningitis and severe forms of disseminated TB in children [3]. In a case-controlled trial in Argentina to determine the efficacy of BCG vaccination against TB in children under the age of 6, the vaccine afforded 98 % protection against TB meningitis and miliary TB [4]. Meta-analysis of trial data showed that BCG could prevent 73 % of childhood TB meningitis and 77 % of miliary TB cases disease in children [5]. However, BCG affords variable efficacy against pulmonary TB, the predominant form of the disease, with a median of 50 % protective efficacy [6]. Recent analysis suggests that BCG can, to some extent, protect against initial infection with *M. tuberculosis*, however this only occurs in a small proportion of exposed individuals [7]. Compounding this is the evidence from field trials that protective immunity afforded by BCG is not lifelong and wanes 10–15 years after vaccination [3]. Considering that TB is a chronic disease where more than 90 % of cases are in adolescents and adults, the limited longevity of the vaccine’s protective effect is a significant barrier to TB control.

**Fig. 1:**
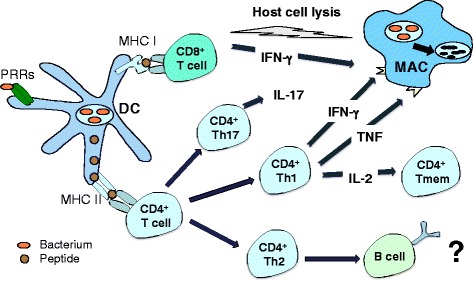
Vaccine-induced immunity to mycobacteria. Dendritic cells (DCs) are activated by vaccine components, such as adjuvants engaging pattern recognition receptors (PRRs), which leads to the presentation of peptide fragments to CD8^+^ and CD4^+^ T-cells. Both Th1 and Th17 CD4^+^ T-cell subsets are associated with protective responses in animal models of *M. tuberculosis* infection, in particular through the stimulation of infected host cells such as macrophages (MAC) to eliminate ingested bacteria. Cytokines (e.g. IL-2) secreted by Th1 CD4^+^ T-cells promote the maintenance of memory T-cell populations (Tmem). Although B cells may be stimulated to produce antibody upon vaccination, they appear to have little role in protection against mycobacteria and are not typically a target of rationally designed vaccines

TB is a complex disease and *M. tuberculosis* has exquisitely adapted to life within the infected host. The disease is characterized by a latent phase, where the organism exists in a form that evades immune clearance, yet is unable to cause active disease [8]. An estimated 2 billion individuals are latently infected with *M. tuberculosis*, representing an enormous reservoir who may reactivate TB later in life. In humans and in animal models, BCG is unable to reduce latent infection and/or prevent reactivation, and as such the development of vaccines that can target latent bacteria would be a significant advance. In addition, new vaccines should induce the type of immunity proposed to protect against *M. tuberculosis* infection. As an obligate intracellular pathogen, a cell-mediated T-cell response is required to contain and ideally eliminate bacteria within infected host cells ([9], Fig. 1). The generation of ‘Th1-like’ CD4^+^ T-cell secreting multiple cytokines are considered to be those required for optimal protective immunity, and most vaccines aim to generate these T-cells at high frequency.

In order to address the deficiencies of BCG, multiple strategies are being used to develop improved TB vaccine candidates. These include boosting BCG-induced immunity, modifying the existing BCG vaccine to improve its effectiveness, or replacing BCG with improved live vaccine candidates. The remainder of this review will describe the current TB vaccine candidates that have advanced to clinical trials, including those developed to prevent TB in children.

## Boosting BCG: subunit vaccines *M. tuberculosis* antigens

It is likely that BCG will remain part of current vaccine schedules, considering its good safety profile and its effectiveness against severe childhood forms of TB. For this reason there is particular interest in the development of subunit vaccines composed of protective *M. tuberculosis* antigens, as these may be used to ‘boost’ the protective response developed by BCG. A handful of vaccines have now entered clinical trials [10], however most of these vaccines make use of a small subset of related secreted antigens. This is despite the fact that *M. tuberculosis* expresses literally hundreds of antigenic targets, including non-secreted proteins [11] and no single mycobacterial antigen is recognised by all *M. tuberculosis*-infected individuals [12–14]. For this reason, all protein vaccines in clinical trials encode multiple antigens to expand the immune recognition of the vaccine (Table 1). In addition, the method of subunit protein delivery is critical for vaccine efficacy and considerable effort has been expended on the identification of potent adjuvants that are nevertheless sufficiently safe and well tolerated for human use [15].

**Table 1:** Tuberculosis vaccine candidates in clinical trials

Category	Vaccine	Clinical trial stage	Trials in children^a^	Vaccine Description
Subunit – protein in adjuvant	M72/AS01	Phase IIb	NCT01098474 (Phase II)	Fusion protein (Mtb39a, Mtb32a) in AS01 adjuvant
	H1/IC31	Phase IIa		Fusion protein (Ag85B, ESAT-6) in IC31 adjuvant
	H4/IC31	Phase II	NCT01861730 (Phase I/II)	Fusion protein (Ag85B, TB10.4) in IC31 adjuvant
	H56/IC31	Phase IIa		Fusion protein (Ag85B, ESAT-6, Rv2660c) in IC31 adjuvant
	ID93/GLA-SE	Phase I		Fusion protein (Rv3619, Rv1813c, Rv3620c, Rv2608) in GLA-SE adjuvant
Subunit – viral vectors	MVA85A	Phase IIb	NCT00953927 (Phase IIb)	Replication-deficient vaccinia Ankara virus expressing Ag85A
	Ad5Ag85A			Replication-deficient adenovirus-5 expressing Ag85A
	AD35.TB-S		NCT01198366 (Phase II)	Replication-defective adenovirus-35 expressing Ag85A, Ag85B, TB10.4
Recombinant BCG	rBCG30	Phase I (discontinued)		Recombinant BCG over-expressing Ag85B
	VPM1002	Phase IIa	NCT01479972 (Phase I) NCT02391415 (Phase IIa)	Recombinant BCG deleted of *ureC* gene and expressing *L. monocytogenes* LLO
	AERAS-422	Phase I (discontinued)		Recombinant BCG expressing Ag85A, Ag85B, Rv3407
Attenuated *M. tuberculosis*	MTBVAC	Phase IIa	NCT02729571 (Phase I)	Live attenuated *M. tuberculosis* deleted of *phoP* and *fadD26* genes
Inactivated Mycobacteria	Dar-901	Phase I		Heat-inactivated *Mycobacterium obuense*

^a^ Clinical trial identifier as taken from www.clinicaltrials.gov

The most advanced fusion protein candidate in terms of clinical development is **M72**/**AS01**. The vaccine is a fusion of two mycobacterial proteins, Mtb39a and Mtb32a, delivered in the AS01 adjuvant, which includes the immune-stimulants 3-*O*-desacyl-4′-monophosphoryl lipid A (MPL) and *Quillaja saponaria* fraction 1 (QS21) combined with liposomes (AS01) [16, 17]. In a Phase I/II trial the vaccine was well tolerated and induced high levels of polyfunctional M72-specific CD4^+^ T-cell and persistent antibody responses [17]. Numerous Phase II trials in adolescents [18], adults [19] or BCG-vaccinated infants [20] demonstrated the generation of polyfunctional CD4^+^ T-cells, augmented humoral responses and no adverse events associated with the vaccine. Importantly, immune responses and vaccine safety were not altered when the vaccine was delivered to infants together with Expanded-Programme-on-Immunization (EPI) vaccines [20]. The vaccine was shown to retain immunogenicity in HIV^+^ subjects on anti-retroviral therapy [21] and has recently entered a Phase IIb proof of concept efficacy study in latently-infected adults (ClinicalTrials.gov Identifier: NCT01755598).

A number of fusion protein vaccines, based on the secreted Ag85B protein of *M. tuberculosis*, are currently under evaluation in humans. **Hybrid 1**/**IC31** comprises Ag85B fused with the early secretory antigenic target 6 (ESAT6), an immunogenic antigen absent from BCG [22]. The fusion protein is adjuvanted with IC31, a 2-component adjuvant comprising an 11-mer antibacterial peptide (KLK) and a synthetic oligodeoxynucleotide (ODN1a), a Toll-like receptor 9 agonist [23]. In Phase I and II trials the vaccine has demonstrated strong, sustained generation of T-cell responses in naïve volunteers [24], individuals previously BCG vaccinated/*M. tuberculosis* infected [25] and HIV^+^ individuals [26]. **Hybrid 4**/**IC31** (AERAS-404) includes the TB10.4 antigen instead of ESAT-6, due to the latter being a component of the Quantiferon Gold diagnostic test for *M. tuberculosis* infection. Hybrid 4/IC31 induced persistent polyfunctional CD4^+^ T-cell responses in adults [27] and the vaccine’s ability to prevent infection with *M. tuberculosis* in adolescents is currently under evaluation (NCT02075203). **Hybrid 56**/**IC31** (AERAS-456) is a modified version of Hybrid 1 that includes Rv2660c, an antigen strongly recognized by the immune response of latent TB patients [28]. The vaccine was shown to protect non-human primates against reactivation of latent *M. tuberculosis* infection [29] and was immunogenic when delivered to healthy adults with or without previous *M. tuberculosis* infection [30]. An ongoing Phase I trial will assess the safety and immunogenicity of Hybrid 56/IC31 in HIV-negative subjects recently treated for drug-susceptible pulmonary TB (NCT02375698) and in BCG-primed infants (NCT01861730).

**ID93**/**GLA**-**SE** is a fusion of four unique antigens (Rv2608, Rv3619, Rv1813, Rv3620) identified during a screen for human anti-mycobacterial T-cell antigens [31]. The vaccine is formulated with the TLR adjuvant glucopyranosyl lipid adjuvant-stable emulsion (GLA-SE) and has shown good protective efficacy in a number of pre-clinical animal models, including mice and guinea pigs, with the generation of polyfunctional T-cell subsets [32]. No clinical data on ID93/GLA-SE has been reported; however, the vaccine is undergoing safety/immunogenicity testing in healthy volunteers (Phase I, NCT01599897, NCT01927159) and in patients following successful completion of TB treatment (NCT02465216).

## Boosting BCG: recombinant viral vectors

Viral vectors have been extensively studied as vaccines to control many pathogens, due to their ability to induce robust cellular and humoral immune responses [33]. The eradication of smallpox by the vaccinia virus has focused attention on the use of poxviruses as vaccine vectors, in particular modified vaccinia virus Ankara (MVA) [33]. **MVA85A** (**AERAS**-**485**), in which the *M. tuberculosis* Ag85A protein is expressed by MVA, was the first TB vaccine to enter human trials [34]. The vaccine, which has been tested in multiple Phase I/IIa studies in adults, adolescents, children and infants, was shown to be well tolerated and induce diverse vaccine-specific T-cell responses [35]. However, in a Phase IIb efficacy trial to test a BCG-prime, MVA85A-boost regimen in BCG-vaccinated South African infants, the vaccine did not provide improved protective efficacy against *M. tuberculosis* infection or disease [36]. An additional Phase IIb trial assessing the efficacy in healthy adults infected with HIV in South Africa and Senegal demonstrated significant T-cell responses induced by the vaccine but there was no improved efficacy against *M. tuberculosis* infection or disease in the MVA85A group compared to placebo [37]. Although the consistency of the pre-clinical animal data supporting this vaccine has been questioned [38], the vaccine has been instrumental in the development of large-scale clinical efficacy trials of vaccines against TB and developing protocols for defining correlates of TB vaccine-induced protection in humans [35].

Replication-deficient adenoviral vectors are an additional class of vaccine vectors that are being utilised for recombinant antigen delivery [33]. **AdAg85A** is an adenoviral serotype 5 vector expressing the *M. tuberculosis* Ag85A protein. In preclinical animal models the vaccine provided optimal protective efficacy when delivered mucosally, in particular the boosting of prior BCG immunization [39]. The vaccine has been tested in humans after intramuscular delivery, however the study was terminated for undefined reasons (NCT00800670). One important issue with adenoviral vectors is the observation that pre-existing Ad5 antibodies have been shown to correlate with Ad5-based HIV vaccine failure [40]. However, infants appear to have reduced levels of neutralizing Ad5 antibodies, which suggests these vaccines may be more suitable for pediatric populations [41]. Pre-existing vector-specific immunity has been overcome by use of chimpanzee adenoviruses (ChAds), with a number of ChAds in clinical trials [33]. This includes a ChAd vector expressing *M. tuberculosis* Ag85A (**ChAdOx185A**), which is being assessed together with a MVA85A boost in adults (NCT01829490). **AD35.TB**-**S** (AERAS-402) is a replication-deficient serotype 35 adenovirus that encodes a fusion of 3 *M. tuberculosis* (Ag85A, Ag85B, TB10.4) and has been assessed in BCG-vaccinated infants and adults, as well as HIV^+^ individuals [42–44]. The vaccine induced polyfunctional CD4^+^/CD8^+^ T-cell and antibody responses to the encoded vaccine antigens, yet induced a modest level of Ad35 antibodies [42–44].

## Improving BCG: recombinant forms of BCG

Despite the limitations of BCG described above, the vaccine does induce some level of protection against childhood forms of TB, and BCG vaccination can reduce mortality in children due to other diseases [45]. Therefore, a major focus of TB vaccine development programs is the development of modified forms of BCG to improve the protective efficacy of the vaccine (reviewed in [46]). Of the numerous recombinant BCG strains developed, only three have undergone human trials. **rBCG30**, which overexpresses the immunodominant Ag85B protein, demonstrated improved efficacy in *M. tuberculosis*-infected guinea pigs, in terms of reduced bacterial loads and improved survival compared to BCG-only vaccinated animals [47]. Although the vaccine was demonstrated to be safe and immunogenic in a Phase I clinical trial in adults, the vaccine is not being pursued further [48]. An alternative strategy was taken in the development of **VPM1002**, a recombinant BCG expressing the *Listeria monocytogenes* enzyme listeriolysin O (LLO) and deleted of the *ureC* gene, in order to facilitate LLO function. Listeriolysin perforates the phagosomal membrane, allowing leakage of enzymes and bacterial components into the cytoplasm and increased apoptosis of the infected cell, resulting in enhanced CD4^+^ and CD8^+^ T-cell responses [49]. The vaccine candidate has completed a Phase I trial in infants (NCT01479972), and is currently being assessed for safety and immunogenicity in HIV-exposed newborns (ClinicalTrials.gov Identifier: NCT02391415).

A dual strategy of antigen overexpression and immune modulation was employed in the development of **AERAS**-**422**, a BCG strain expressing the pore-forming perfringolysin of *Clostridium perfringens* and selected immunodominant antigens expressed by *M. tuberculosis* during active infection (Ag85A and Ag85B) and reactivation of latent infection (Rv3407) [50]. In a Phase I trial in adults, the vaccine induced strong antigen-specific T-cell responses; however, two of eight vaccinees developed varicella zoster virus (VZV) reactivation, resulting in the discontinuation of the vaccine’s development [51].

## Replacing BCG: live attenuated mycobacterial strains

An alternative approach to TB vaccine design is to use live mycobacterial strains to replace BCG in the childhood vaccine schedule. When compared to virulent *M. tuberculosis* isolates, more than one hundred genes are absent in the BCG genome [52], and a subset of these genes may be important protective antigens. Therefore, a rationally attenuated form of *M. tuberculosis* may more closely ‘mimic’ the immune repertoire generated by natural infection. **MTBVAC** is the first live-attenuated *M. tuberculosis*-based vaccine to undergo testing in humans. **MTBVAC** contains two independent stable deletion mutations in the virulence genes *phoP* and *fadD26*, without the inclusion of antibiotic resistance markers, thus fulfilling the second Geneva Consensus requirements for progression of live mycobacterial vaccines to human trials [53, 54]. The vaccine afforded significant protection against *M. tuberculosis* in pre-clinical models [55] and induced markedly enhanced T-cell immunity compared to the BCG vaccine in mice [56]. In a Phase I clinical trial **MTBVAC** did not induce any serious adverse events and elicited the generation of polyfunctional CD4^+^ central memory T-cells in vaccinees [57]. Encouragingly, vaccine safety and immunogenicity is currently being assessed in newborns in a Phase I trial (NCT02729571).

Inactivated whole-cell mycobacterial strains are also being assessed, although mainly as post-infection/immunotherapeutic vaccines, with the aim of preventing reactivation and/or shortening the course of drug-treatment for TB. The immunotherapeutic potential of these vaccines has been reviewed elswehere [58] and will not be discussed here. One inactivated vaccine, termed **Dar**-**901**, is under evaluation as a preventative TB vaccine. Dar-901 consists of the heat-inactivated non-tuberculous *M. obuense* and is part of a Phase I clinical trial for safety and immunogenicity healthy adults (NCT02063555), and is currently recruiting for a Phase II trial as a booster to prevent TB in adoloscents (NCT02712424).

## Challenges of TB vaccine development

A number of challenges remain before a new vaccine can be introduced to either complement or replace the existing BCG vaccine. Although there is an appreciation that multiple antigens should be included into subunit vaccine to broaden the immune response generated, many candidates use single antigens—in particular Ag85A, which in a recent study was not strongly recognized by the immune response of TB patients [14]. Unlike viruses, which tend express a limited number of antigenic targets, the antigen repertoire of mycobacteria is broad and includes poorly expressed and cryptic epitopes that may contribute to protection [59]. Therefore new vaccine candidates should ideally express a selection of antigens that are strongly recognised by the human immune response during all stages of the TB life cycle. In addition, assessment of antigen immune recognition has typically been performed in adult TB patients, rather than children [14] and it is possible that the infant immune system may display differential recognition of antigens. Compounding this is the fact that most clinical trials of TB vaccine have been performed in adolescent or adult populations (Table 1), thus limiting our knowledge of vaccine-induced immunity in children.

An additional consideration is the lack of defined immune correlates of protection against *M. tuberculosis* infection. Certain immune responses appear to correlate with protection against *M. tuberculosis* in animal models, such as CD4^+^ T-cell secreting mutiple cytokines, termed ‘polyfunctional T’ cells [60]. However, the presence of polyfunctional T-cells in either MVA85A-vaccinated adults or BCG-vaccinated infants did not correlate with protection against TB in humans [36, 61]. While this may be a function of the particular vaccine tested, it does suggest that a greater breadth of immune paramaters should be examined as potential ‘biomarkers’ of protection, including non-conventional T-cells and components of the innate immune response [9]. Importantly, while the mouse is the major animal model used for testing of TB vaccines, the relative contribution of immune cell subsets may differ between the mouse, humans and other models such as non-human primates [62]. This makes it difficult to extrapolate findings from animal models to humans. While a human challenge model for TB has been proposed [63], the curent model relies on use of the attenuated BCG vaccine as the challenge organism, which lacks important TB vaccine antigens, and further optimisation is required to overcome the low level of recovery of the challenge strain [64]. However, such a model would provide an important tool for TB vaccine research, considering the expense of large phase IIb and phase III clinical trials to determine vaccine efficacy for a chronic infection such as TB, together with the lack of markers of protective immunity in humans.

## Conclusions

The past decade has seen major advances in the development of TB vaccines, with a number of vaccines now in clinical trials (Table 1) and one vaccine having completed Phase IIb assessment of efficacy [36]. However, no vaccine has demonstrated improved protective efficacy in humans compared to the existing BCG vaccine, and the immunological parameters required for effective protective efficacy in humans are not known. This suggests that innovative and novel TB vaccine approaches are required and these vaccines should elicit immune responses that differ from those candidates that have already been evaluated in humans. Effective control of TB transmission will require the delivery of a vaccine that can block/limit *M. tuberculosis* infection during initial exposure to the pathogen, which in endemic areas would be during early childhood, and thus the testing of new candidates in infant populations should be a priority.

## Abbreviations

BCG: Bacille Calmette-Guérin
ChAds: Chimpanzee adenoviruses
GLA-SE: Glucopyranosyl lipid adjuvant-stable emulsion
MPL: 3-*O*-desacyl-4′-monophosphoryl lipid A (MPL)
MVA: Modified vaccinia virus Ankara
TB: Tuberculosis

## References

[1] Organization WH. Global tuberculosis report 2015. Only available online: http://www.who.int/tb/publications/global_report/. 2015.

[2] Perez-Velez CM, Marais BJ (2012). Tuberculosis in children. N Engl J Med.

[3] Mangtani P, Abubakar I, Ariti C, Beynon R, Pimpin L, Fine PE (2014). Protection by BCG vaccine against tuberculosis: a systematic review of randomized controlled trials. Clin Infect Dis.

[4] Miceli I, De Kantor IN, Colaiacovo D, Peluffo G, Cutillo I, Gorra R (1988). Evaluation of the effectiveness of BCG vaccination using the case–control method in Buenos Aires, Argentina. Int J Epidemiol.

[5] Trunz BB, Fine P, Dye C (2006). Effect of BCG vaccination on childhood tuberculous meningitis and miliary tuberculosis worldwide: a meta-analysis and assessment of cost-effectiveness. Lancet.

[6] Colditz GA, Brewer TF, Berkey CS, Wilson ME, Burdick E, Fineberg HV (1994). Efficacy of BCG vaccine in the prevention of tuberculosis. Meta-analysis of the published literature. JAMA.

[7] Roy A, Eisenhut M, Harris RJ, Rodrigues LC, Sridhar S, Habermann S (2014). Effect of BCG vaccination against *Mycobacterium tuberculosis* infection in children: systematic review and meta-analysis. BMJ.

[8] Dheda K, Barry CE, Maartens G (2016). Tuberculosis. Lancet.

[9] Nunes-Alves C, Booty MG, Carpenter SM, Jayaraman P, Rothchild AC, Behar SM (2014). In search of a new paradigm for protective immunity to TB. Nat Rev Microbiol.

[10] Kaufmann SH, Lange C, Rao M, Balaji KN, Lotze M, Schito M (2014). Progress in tuberculosis vaccine development and host-directed therapies-a state of the art review. Lancet Respir Med.

[11] Pinto R, Leotta L, Shanahan ER, West NP, Leyh TS, Britton W (2013). Host cell-induced components of the sulfate assimilation pathway are major protective antigens of *Mycobacterium tuberculosis*. J Infect Dis.

[12] Roche PW, Triccas JA, Avery DT, Fifis T, Billman-Jacobe H, Britton WJ (1994). Differential T cell responses to mycobacteria-secreted proteins distinguish vaccination with Bacille Calmette-Guerin from infection with *Mycobacterium tuberculosis*. J Infect Dis.

[13] Sutherland JS, Lalor MK, Black GF, Ambrose LR, Loxton AG, Chegou NN (2013). Analysis of host responses to *Mycobacterium tuberculosis* antigens in a multi-site study of subjects with different TB and HIV infection states in sub-Saharan Africa. PLoS ONE.

[14] Carpenter C, Sidney J, Kolla R, Nayak K, Tomiyama H, Tomiyama C (2015). A side-by-side comparison of T cell reactivity to fifty-nine *Mycobacterium tuberculosis* antigens in diverse populations from five continents. Tuberculosis.

[15] Agger EM. Novel adjuvant formulations for delivery of anti-tuberculosis vaccine candidates. Adv Drug Deliv Rev. 2015;17. 10.1016/j.addr.2015.11.012 PMC4870161 26596558

[16] Skeiky YA, Lodes MJ, Guderian JA, Mohamath R, Bement T, Alderson MR (1999). Cloning, expression, and immunological evaluation of two putative secreted serine protease antigens of *Mycobacterium tuberculosis*. Infect Immun.

[17] Leroux-Roels I, Forgus S, De Boever F, Clement F, Demoitie MA, Mettens P (2013). Improved CD4(+) T cell responses to *Mycobacterium tuberculosis* in PPD-negative adults by M72/AS01 as compared to the M72/AS02 and Mtb72F/AS02 tuberculosis candidate vaccine formulations: a randomized trial. Vaccine.

[18] Penn-Nicholson A, Geldenhuys H, Burny W, van der Most R, Day CL, Jongert E (2015). Safety and immunogenicity of candidate vaccine M72/AS01E in adolescents in a TB endemic setting. Vaccine.

[19] Montoya J, Solon JA, Cunanan SR, Acosta L, Bollaerts A, Moris P (2013). A randomized, controlled dose-finding Phase II study of the M72/AS01 candidate tuberculosis vaccine in healthy PPD-positive adults. J Clin Immunol.

[20] Idoko OT, Owolabi OA, Owiafe PK, Moris P, Odutola A, Bollaerts A (2014). Safety and immunogenicity of the M72/AS01 candidate tuberculosis vaccine when given as a booster to BCG in Gambian infants: an open-label randomized controlled trial. Tuberculosis.

[21] Kumarasamy N, Poongulali S, Bollaerts A, Moris P, Beulah FE, Ayuk LN (2016). A Randomized, Controlled Safety, and Immunogenicity Trial of the M72/AS01 Candidate Tuberculosis Vaccine in HIV-Positive Indian Adults. Medicine.

[22] Weinrich Olsen A, Van Pinxteren LA, Meng Okkels L, Birk Rasmussen P, Andersen P (2001). Protection of mice with a tuberculosis subunit vaccine based on a fusion protein of antigen 85B and ESAT-6. Infect Immun.

[23] Aichinger MC, Ginzler M, Weghuber J, Zimmermann L, Riedl K, Schutz G (2011). Adjuvating the adjuvant: facilitated delivery of an immunomodulatory oligonucleotide to TLR9 by a cationic antimicrobial peptide in dendritic cells. Vaccine.

[24] Van Dissel JT, Arend SM, Prins C, Bang P, Tingskov PN, Lingnau K (2010). Ag85B-ESAT-6 adjuvanted with IC31 promotes strong and long-lived *Mycobacterium tuberculosis* specific T cell responses in naive human volunteers. Vaccine.

[25] Van Dissel JT, Soonawala D, Joosten SA, Prins C, Arend SM, Bang P (2011). Ag85B-ESAT-6 adjuvanted with IC31(R) promotes strong and long-lived *Mycobacterium tuberculosis* specific T cell responses in volunteers with previous BCG vaccination or tuberculosis infection. Vaccine.

[26] Reither K, Katsoulis L, Beattie T, Gardiner N, Lenz N, Said K (2014). Safety and immunogenicity of H1/IC31(R), an adjuvanted TB subunit vaccine, in HIV-infected adults with CD4+ lymphocyte counts greater than 350 cells/mm3: a phase II, multi-centre, double-blind, randomized, placebo-controlled trial. PLoS ONE.

[27] Geldenhuys H, Mearns H, Miles DJ, Tameris M, Hokey D, Shi Z (2015). The tuberculosis vaccine H4:IC31 is safe and induces a persistent polyfunctional CD4 T cell response in South African adults: A randomized controlled trial. Vaccine.

[28] He H, Yang H, Deng Y (2015). *Mycobacterium tuberculosis* dormancy-associated antigen of Rv2660c induces stronger immune response in latent *Mycobacterium tuberculosis* infection than that in active tuberculosis in a Chinese population. Eur J Clin Microbiol Infect Dis.

[29] Lin PL, Dietrich J, Tan E, Abalos RM, Burgos J, Bigbee C (2012). The multistage vaccine H56 boosts the effects of BCG to protect cynomolgus macaques against active tuberculosis and reactivation of latent *Mycobacterium tuberculosis* infection. J Clin Invest.

[30] Luabeya AK, Kagina BM, Tameris MD, Geldenhuys H, Hoff ST, Shi Z (2015). First-in-human trial of the post-exposure tuberculosis vaccine H56:IC31 in *Mycobacterium tuberculosis* infected and non-infected healthy adults. Vaccine.

[31] Bertholet S, Ireton GC, Kahn M, Guderian J, Mohamath R, Stride N (2008). Identification of human T cell antigens for the development of vaccines against *Mycobacterium tuberculosis*. J Immunol.

[32] Baldwin SL, Bertholet S, Reese VA, Ching LK, Reed SG, Coler RN (2012). The importance of adjuvant formulation in the development of a tuberculosis vaccine. J Immunol.

[33] Ewer K, Lambe T, Rollier C, Spencer A, Hill A, Dorrell L (2016). Viral vectors as vaccine platforms: from immunogenicity to impact. Curr Opin Immunol.

[34] McShane H, Pathan AA, Sander CR, Keating SM, Gilbert SC, Huygen K (2004). Recombinant modified vaccinia virus Ankara expressing antigen 85A boosts BCG-primed and naturally acquired antimycobacterial immunity in humans. Nature Med.

[35] O’Shea MK, McShane H (2016). A review of clinical models for the evaluation of human TB vaccines. Hum Vacc Immunother.

[36] Tameris MD, Hatherill M, Landry BS, Scriba TJ, Snowden MA, Lockhart S (2013). Safety and efficacy of MVA85A, a new tuberculosis vaccine, in infants previously vaccinated with BCG: a randomised, placebo-controlled phase 2b trial. Lancet.

[37] Ndiaye BP, Thienemann F, Ota M, Landry BS, Camara M, Dieye S (2015). Safety, immunogenicity, and efficacy of the candidate tuberculosis vaccine MVA85A in healthy adults infected with HIV-1: a randomised, placebo-controlled, phase 2 trial. Lancet Respir Med.

[38] Kashangura R, Sena ES, Young T, Garner P (2015). Effects of MVA85A vaccine on tuberculosis challenge in animals: systematic review. Int J Epidemiol.

[39] Xing Z, McFarland CT, Sallenave JM, Izzo A, Wang J, McMurray DN (2009). Intranasal mucosal boosting with an adenovirus-vectored vaccine markedly enhances the protection of BCG-primed guinea pigs against pulmonary tuberculosis. PLoS ONE.

[40] Buchbinder SP, Mehrotra DV, Duerr A, Fitzgerald DW, Mogg R, Li D (2008). Efficacy assessment of a cell-mediated immunity HIV-1 vaccine (the Step Study): a double-blind, randomised, placebo-controlled, test-of-concept trial. Lancet.

[41] Appaiahgari MB, Pandey RM, Vrati S (2007). Seroprevalence of neutralizing antibodies to adenovirus type 5 among children in India: implications for recombinant adenovirus-based vaccines. Clin Vaccine Immunol.

[42] Churchyard GJ, Snowden MA, Hokey D, Dheenadhayalan V, McClain JB, Douoguih M (2015). The safety and immunogenicity of an adenovirus type 35-vectored TB vaccine in HIV-infected, BCG-vaccinated adults with CD4(+) T cell counts >350 cells/mm(3). Vaccine.

[43] Kagina BM, Tameris MD, Geldenhuys H, Hatherill M, Abel B, Hussey GD (2014). The novel tuberculosis vaccine, AERAS-402, is safe in healthy infants previously vaccinated with BCG, and induces dose-dependent CD4 and CD8 T cell responses. Vaccine.

[44] Walsh DS, Owira V, Polhemus M, Otieno L, Andagalu B, Ogutu B (2016). Adenovirus type 35-vectored tuberculosis vaccine has an acceptable safety and tolerability profile in healthy, BCG-vaccinated, QuantiFERON((R))-TB Gold (+) Kenyan adults without evidence of tuberculosis. Vaccine.

[45] Kleinnijenhuis J, Van Crevel R, Netea MG (2015). Trained immunity: consequences for the heterologous effects of BCG vaccination. Trans R Soc Trop Med Hyg.

[46] Triccas JA (2010). Recombinant BCG, as a vaccine vehicle to protect against tuberculosis. Bioengineered bugs.

[47] Horwitz MA, Harth G, Dillon BJ, Maslesa-Galic S (2000). Recombinant bacillus calmette-guerin (BCG) vaccines expressing the *Mycobacterium tuberculosis* 30-kDa major secretory protein induce greater protective immunity against tuberculosis than conventional BCG vaccines in a highly susceptible animal model. Proc Natl Acad Sci U S A.

[48] Hoft DF, Blazevic A, Abate G, Hanekom WA, Kaplan G, Soler JH (2008). A new recombinant bacille Calmette-Guerin vaccine safely induces significantly enhanced tuberculosis-specific immunity in human volunteers. J Infect Dis.

[49] Farinacci M, Weber S, Kaufmann SH (2012). The recombinant tuberculosis vaccine rBCG DeltaureC::hly(+) induces apoptotic vesicles for improved priming of CD4(+) and CD8(+) T cells. Vaccine.

[50] Sun R, Skeiky YA, Izzo A, Dheenadhayalan V, Imam Z, Penn E (2009). Novel recombinant BCG expressing perfringolysin O and the over-expression of key immunodominant antigens; pre-clinical characterization, safety and protection against challenge with *Mycobacterium tuberculosis*. Vaccine.

[51] Hoft DF, Blazevic A, Selimovic A, Turan A, Tennant J, Abate G (2016). Safety and Immunogenicity of the Recombinant BCG Vaccine AERAS-422 in Healthy BCG-naïve Adults: A Randomized, Active-controlled, First-in-human Phase 1 Trial. EBioMedicine.

[52] Brosch R, Gordon SV, Garnier T, Eiglmeier K, Frigui W, Valenti P (2007). Genome plasticity of BCG and impact on vaccine efficacy. Proc Natl Acad Sci U S A.

[53] Arbues A, Aguilo JI, Gonzalo-Asensio J, Marinova D, Uranga S, Puentes E (2013). Construction, characterization and preclinical evaluation of MTBVAC, the first live-attenuated *M. tuberculosis*-based vaccine to enter clinical trials. Vaccine.

[54] Walker KB, Brennan MJ, Ho MM, Eskola J, Thiry G, Sadoff J (2010). The second Geneva Consensus: Recommendations for novel live TB vaccines. Vaccine.

[55] Scriba TJ, Kaufmann SH, Lambert PH, Sanicas M, Martin C, Neyrolles O (2016). Vaccination against tuberculosis with whole cell mycobacterial vaccines. J Infect Dis.

[56] Nambiar JK, Pinto R, Aguilo JI, Takatsu K, Martin C, Britton WJ (2012). Protective immunity afforded by attenuated, PhoP-deficient *Mycobacterium tuberculosis* is associated with sustained generation of CD4+ T-cell memory. Eur J Immunol.

[57] Spertini F, Audran R, Chakour R, Karoui O, Steiner-Monard V, Thierry AC (2015). Safety of human immunisation with a live-attenuated *Mycobacterium tuberculosis* vaccine: a randomised, double-blind, controlled phase I trial. Lancet Respir Med.

[58] Groschel MI, Prabowo SA, Cardona PJ, Stanford JL, van der Werf TS (2014). Therapeutic vaccines for tuberculosis--a systematic review. Vaccine.

[59] Woodworth JS, Aagaard CS, Hansen PR, Cassidy JP, Agger EM, Andersen P (2014). Protective CD4 T cells targeting cryptic epitopes of *Mycobacterium tuberculosis* resist infection-driven terminal differentiation. J Immunol.

[60] Lindenstrom T, Knudsen NP, Agger EM, Andersen P (2013). Control of chronic mycobacterium tuberculosis infection by CD4 KLRG1- IL-2-secreting central memory cells. J Immunol.

[61] Kagina BM, Abel B, Scriba TJ, Hughes EJ, Keyser A, Soares A (2010). Specific T cell frequency and cytokine expression profile do not correlate with protection against tuberculosis after bacillus Calmette-Guerin vaccination of newborns. Am J Respir Crit Care Med.

[62] Chen CY, Huang D, Wang RC, Shen L, Zeng G, Yao S (2009). A critical role for CD8 T cells in a nonhuman primate model of tuberculosis. PLoS Pathog.

[63] Minassian AM, Satti I, Poulton ID, Meyer J, Hill AV, McShane H (2012). A human challenge model for Mycobacterium tuberculosis using *Mycobacterium bovis* bacille Calmette-Guerin. J Infect Dis.

[64] Minhinnick A, Harris S, Wilkie M, Peter J, Stockdale L, Manjaly-Thomas ZR (2016). Optimization of a Human Bacille Calmette-Guerin Challenge Model: A Tool to Evaluate Antimycobacterial Immunity. J Infect Dis.

